# An Atypical Adverse Local Tissue Reaction Seen With Metal-On-Metal Total Hip Arthroplasty Utilizing Polyethylene Liners With Metal Inlays

**DOI:** 10.1016/j.artd.2023.101106

**Published:** 2023-03-09

**Authors:** Hemant Reddy, Yoav Zvi, Mitchell Weiser

**Affiliations:** Department of Orthopedic Surgery, Montefiore Medical Center/Albert Einstein College of Medicine, Bronx, NY, USA

**Keywords:** Metal on metal THA, Metal inlay polyethylene liner, Revision THA

## Abstract

Adverse local tissue reaction (ALTR) is a frequently described, although uncommon, complication of metal-on-metal total hip arthroplasty. Here in we report on 2 patients with unique metal-inlay polyethylene liners who suffered from ALTR that required revision arthroplasty. In 2 of 3 cases the femoral trunnion was noted to have minimal corrosion and the stem was salvaged with a titanium adapter. In one case there was catastrophic femoral stem failure and an extended trochanteric osteotomy was required to remove the unique lateral flare stem. The surgeon must pay special attention when scrutinizing radiographs to identify a metal inlay polyethylene liner and when performing revision arthroplasty to prevent greater trochanter fracture in a patient who likely already has compromised abductor function due to ALTR.

## Introduction

Adverse local tissue reaction (ALTR) is a morbid complication that has historically been seen with metal-on-metal (MoM) total hip arthroplasties (THAs). ALTRs are primarily lymphocytic reactions to metal debris that may cause aseptic loosening, osteolysis and local soft-tissue destruction resulting in pain, instability, and significant morbidity [1]. ALTR can progress further resulting in formation of a pseudotumor, which is a granulomatous or destructive cystic lesion than resembles tumor, but is not infection or neoplasm [2]. ALTRs are not limited to solely MoM-THA and can be even seen with metal-on-polyethylene implants due to corrosion at the morse taper of femoral implant trunnion, a process known as mechanically assisted crevice corrosion [3,4].

During the recall of many monoblock first-generation MoM-THAs that were causing early failure, acetabular components with polyethylene liners with a metal inlay were adopted as an alternative to retain the excellent wear properties of second-generation MoM-THAs. Additionally, in the event of incorrect cup placement, the polyethylene metal inlays allow for polyethylene impingement rather than MoM impingement [5]. The Metasul metal bearing system (Zimmer, Warsaw, IN) included a metal inlay polyethylene liner with a satisfactory survivorship up to 15 years, even in patients younger than 50 years [[6], [7], [8]]. While the Metasul metal bearing system reported good survivability, a similar design was manufactured by Encore Medical/DJO (Austin, TX), the FMP acetabular system. However, there remains a paucity of literature regarding the long-term outcomes of this implant system and the Food and Drug Administration Manufacturer and User Facility Device Experience database has many reports of revision surgery for this system. Herein, we describe 3 cases of 2 patients with primary THA utilizing the FMP acetabular system (Encore Medical/DJO, Austin, TX) with metal inlay polyethylene liners that required revision surgery. These cases are atypical due to the implant of choice which causes metallosis as well as osteolysis from polyethylene wear due to the nature of the metal inlay shell and suboptimal track record of the polyethylene.

## Case histories

Written informed consent for the publication of this case was obtained from both patients.

### Patient 1 – case 1

#### Preoperative care

A 62-year-old man with medical history significant for hypertension, benign prostatic hyperplasia, obstructive sleep apnea, and mitral valve prolapse was found to have recurrent bilateral groin swelling directly after bilateral robotic inguinal hernia surgery. During hernia surgery, black pigmented inguinal lymph nodes were appreciated with pathology consistent with reactive follicles and sinus histiocytes containing pigment. He was referred to our orthopedic clinic for evaluation after magnetic resonance imaging (MRI) revealed lytic pelvic lesions and a soft-tissue mass associated with his bilateral total hip arthroplasties.

He presents with bilateral groin pain, left worse than right for the past 2 years. His pain is exacerbated with activities of deep hip flexion. He ambulates with a cane and is limited to 1-2 blocks. He denies any history of fevers, chills, weight loss, or night sweats. He had undergone staged bilateral primary THA in 2009, 12 years prior, with the FMP acetabular system (Encore Medical/DJO, Austin, TX) utilizing a metal inlay polyethylene liner and DJO Revelation stem system (Austin, TX) and had an uncomplicated postoperative course.

His physical examination is significant for a large firm left groin mass at the level of the superficial inguinal ring. Bilateral hip range of motion was noted at 100 degrees of hip flexion and 30 degrees of abduction and full bilateral abductor strength. Left leg was noted to be 10 mm shorter.

**Figure 1:**
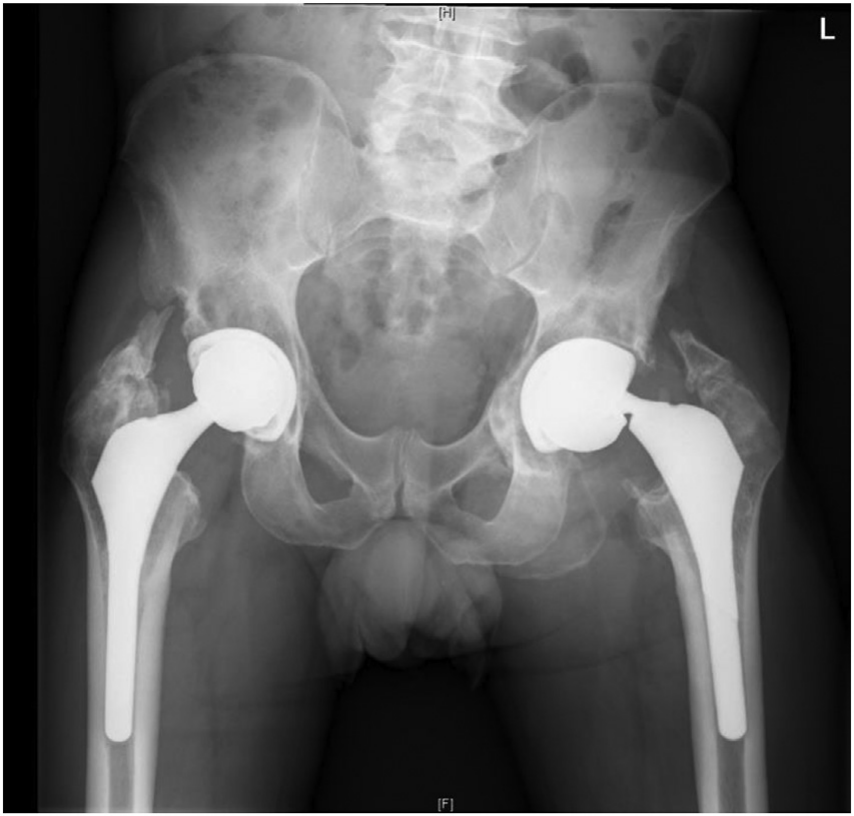
Anteroposterior pelvis radiograph of Case 1. A 62-year-old man with bilateral metal-on-metal total hip arthroplasties (THAs) and impending catastrophic failure of the left THA.

Radiographs at initial presentation were consistent with bilateral MoM-THAs with loosening of the left acetabulum and heterotopic ossification within the abductors (Fig. 1). Metal loss at the inferior aspect of the left femoral neck was present indicating notching of the femoral neck and overall varus alignment. The left acetabular liner was dissociated and rotated greater than 90 degrees. The right MoM-THA was in overall stable alignment with mild retroacetabular lucencies. The erythrocyte sedimentation rate was 13 mm/h and C-reactive protein was 0.6 mg/dL, both within normal limits. Serum cobalt was 10 parts per billion (ppb) and chromium 5.7 ppb, both elevated.

**Figure 2:**
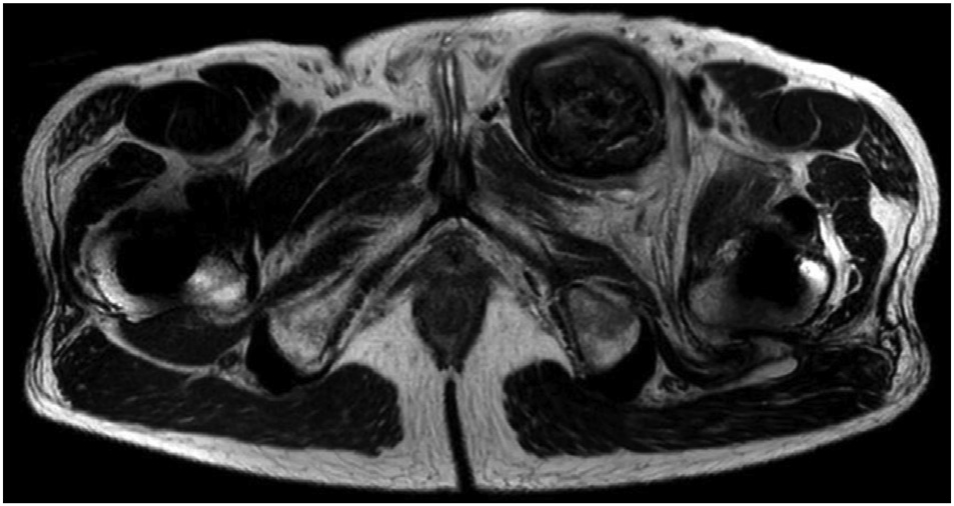
Axial T2 MARS-MRI pelvis with contrast demonstrates pseudotumor formation within the left anterior pelvis and bilateral peritrochanteric fluid signal. MARS = magnetic artifact reduction sequence.

A magnetic artifact reduction sequence-MRI pelvis with contrast was consistent with bilateral retroacetabular osteolysis with the left being worse and compromising the anterior column of the pelvis (Fig. 2). Additionally, a 5.5-cm rounded groin mass medial to the femoral vessels was seen with signal consistent with the presence of metal. The left groin cyst was aspirated under computed tomography guidance and 50 cc of fluid was removed. The automatic white blood cell count was 144,340, but a manual cell count was not obtained. Gram stain was negative, and no growth appeared on final cultures.

### Intraoperative care

**Figure 3:**
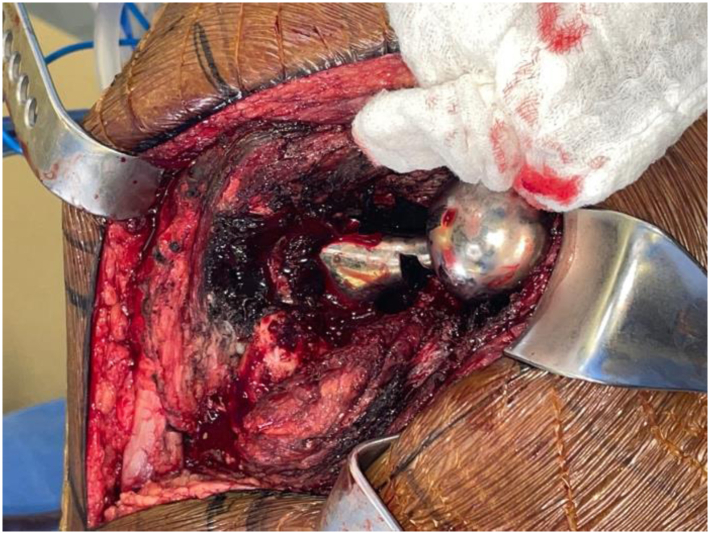
Intraoperative view of case 1’s left revision total hip arthroplasty with almost complete notching of the femoral neck visualized. There is also extensive black staining of the pericapsular tissue.

**Figure 4:**
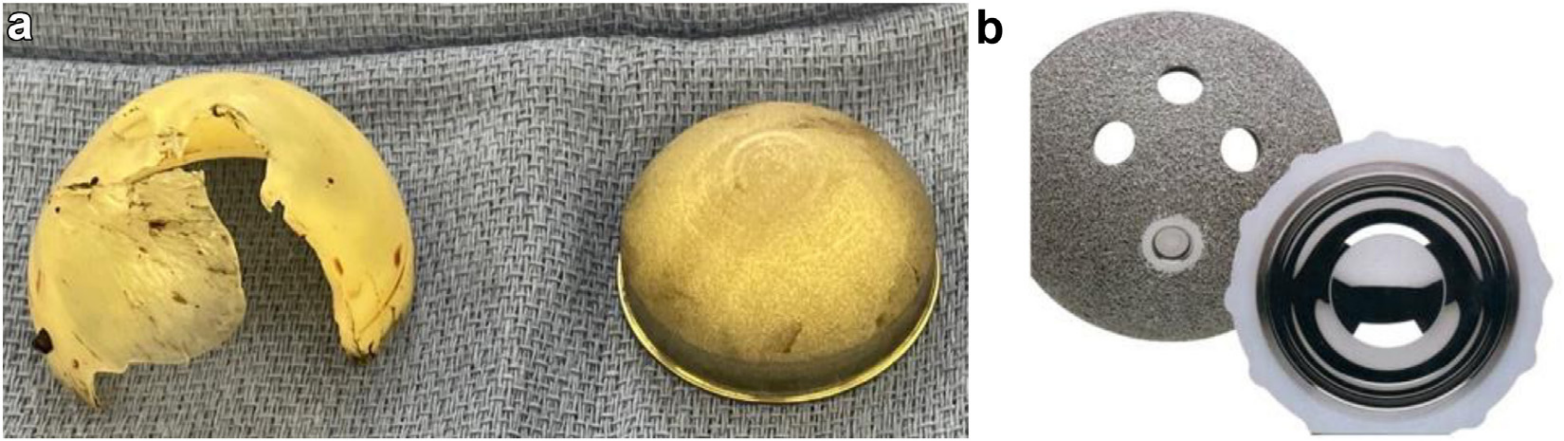
Fractured and intact metal-inlay polyethylene liner. (a) Visualized on the left is the fracture polyethylene liner and the metal inlay on the right. (b) An example of an intact metal-inlay polyethylene liner from the FMP acetabular system (Encore Medical/DJO, Austin, TX, USA) [9].

Given the risk of impending catastrophic failure of the left THA and pseudotumor perforation of the anterior column, he was indicated for left revision THA. A standard posterior approach through the previous incision was utilized. Upon entering the capsule, a massive efflux of black liquid consistent with metallosis was encountered. The capsule and surrounding tissue were completely blackened by metal debris and thoroughly debrided. The hip was dislocated and the femoral implant was stressed and determined to be well fixed. Near-complete notching of the inferior femoral neck was encountered (Fig. 3). The metal inlay polyethylene liner was completely dissociated from the shell and the polyethylene was fractured in several places (Fig. 4a and b). An extended trochanteric osteotomy was performed to expose and debond the femoral stem. An acetabular explant system was used to circumferentially debond the cup with minimal bone loss. Extensive osteolysis of the anterior wall and column and posterior column and ischium were noted, but the superior acetabular dome was intact. Bone defects were filled with cancellous chips and reamed on reverse prior to acetabular cup impaction. A 70-mm Depuy Pinnacle revision shell (Warsaw, IN) was impacted followed by 3 acetabular screws. Cerclage wires were then placed distal to the femoral osteotomy to prevent fracture propagation during femoral preparation. A Depuy Reclaim (Warsaw, IN) modular femoral component with 19 mm × 140 mm distal stem and a 24 mm × 85 mm proximal body was then impacted followed by a Depuy Bimentum (Warsaw, IN) dual mobility construct with 28-mm head and 28/53-mm liner. The final implant was stable with range of motion and the femoral osteotomy was closed with cerclage cables. The incision was then closed in standard layered fashion.

### Postoperative care

Postoperatively, he was made toe-touch weight bearing with trochanteric precautions. His postimmediate postoperative course required 1 unit of packed red blood cell transfusion due to acute anemia but then was discharged home. At the 2-week follow-up, he was noted to have a new nondisplaced fracture line through the greater trochanter which was treated nonoperatively with continued toe-touch weight bearing. At the 6-week follow-up, interval callus formation was appreciated so trochanteric precautions were discontinued. At the 3-month follow-up, his left hip pain was now minimal and he was walking with a cane. A repeat magnetic artifact reduction sequence-MRI pelvis at this time revealed peritrochanteric fluid around his right hip without evidence of pseudotumor or abductor complex destruction and he wanted to proceed with right revision THA.

### Patient 1 – case 2

Six months after left revision THA, he was taken for right revision THA. A standard posterior approach to the hip through the previous incision was performed. Black staining of the pericapsular tissue was again appreciated. There was only minimal corrosion of the trunnion and a stable femoral stem so an extended trochanteric osteotomy and femoral stem explanation was not performed. After acetabular explantation, retroacetabular defects were filled with cancellous bone chips, reamed on reverse and a 64-mm Zimmer Biomet G7 (Warsaw, IN) revision cup was then impacted followed by 4 pelvic screws. A 40-mm Biolox Delta OPTION ceramic femoral head with a titanium sleeve adaptor manufactured by DJO Global (Austin, TX) was then placed. The incision was then closed in standard layered fashion.

**Figure 5:**
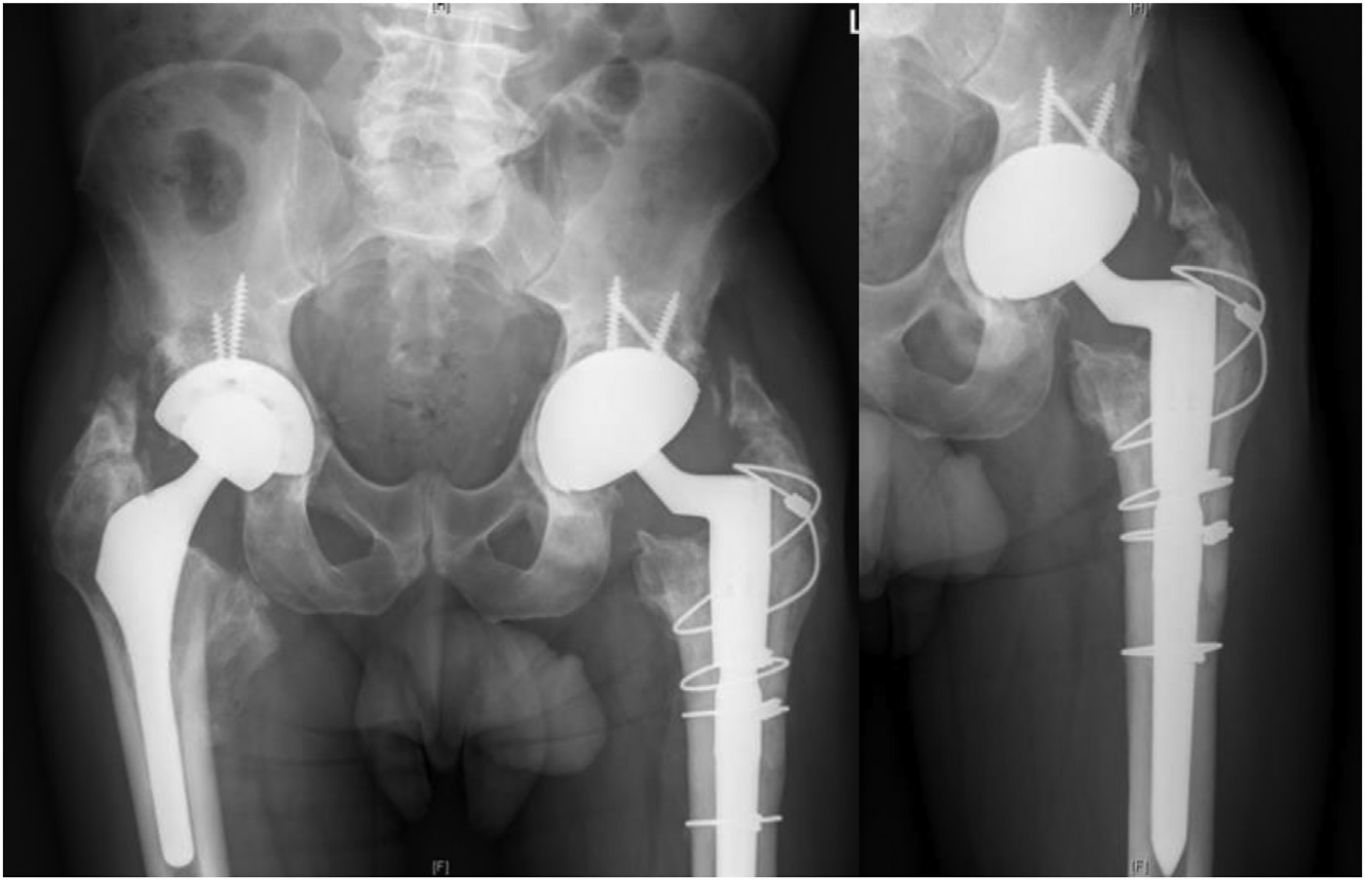
Postoperative radiographs 1 year from left and 6 months from right revision hip arthroplasty.

The acute postoperative course required 1 unit of packed red blood cell transfusion due to acute blood loss anemia but was otherwise uncomplicated. He was subsequently discharged home. At the final follow-up 18 months from the left sided revision and 12 months from right sided revision, he is ambulating well, utilizing a cane occasionally, and only experiencing occasional pain in either hip (Fig. 5). Additionally, his left groin mass had significantly reduced in size.

### Patient 2 – case 3

#### Preoperative care

A 64-year-old man with past medical history notable for hypertension, stroke, coronary artery disease, and kidney transplantation on chronic immunosuppressive medication presented to our clinic for evaluation of persistent right hip pain after previous primary THA in 2006, 12 years prior. He had also previously undergone left hip revision THA 3 years prior by another surgeon for a similar primary MoM-THA implant. He reports 2 months of severe right groin pain and is limited to walking less than 1 block. On examination, he has 90 degrees of hip flexion, tenderness to palpation in the groin and at the greater trochanter, and 4/5 abductor strength. Laboratory test results revealed an erythrocyte sedimentation rate of 3 mm/h and C-reactive protein of 0.8 mg/dL, both within normal limits. Metal ion levels were elevated to 2.2 ppb for cobalt and 2.1 ppb for chromium.

**Figure 6:**
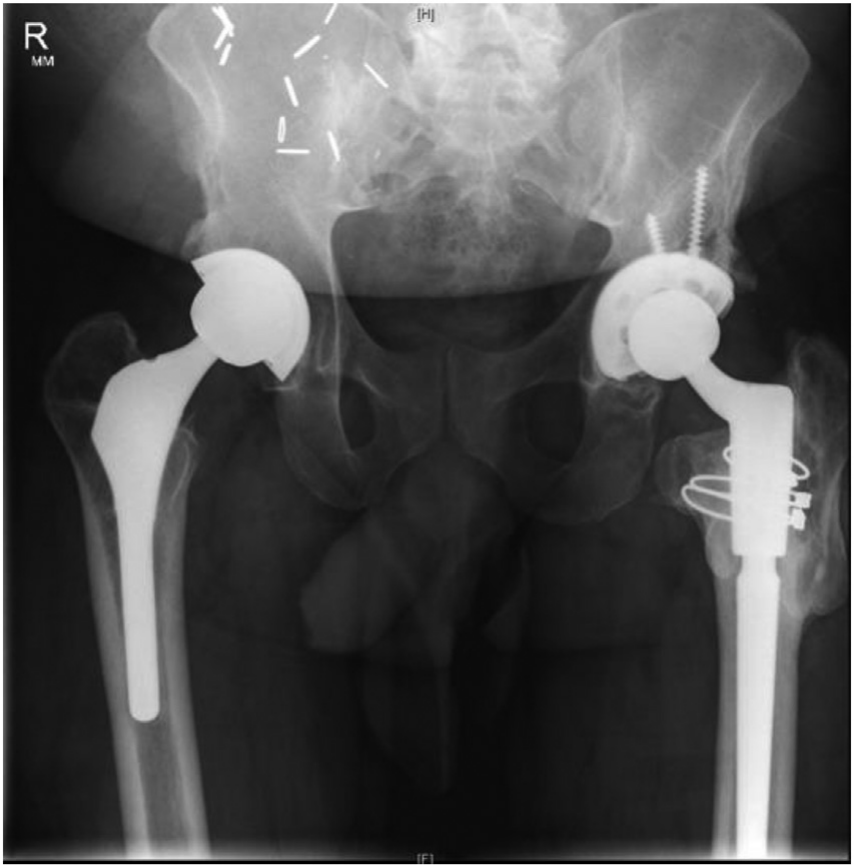
Initial pelvis radiographs for Patient 2-case 3. A 64-year-old man with a right metal-on-metal THA with slight eccentric positioning of the femoral head within the acetabulum. The metal inlay can be visualized on close inspection. A previous revision THA is visualized in the contralateral limb.

Radiographs were consistent with a right MoM-THA with slight eccentric positioning of the femoral head without significant retroacetabular osteolysis (Fig. 6). There is also a revision left THA in good alignment without loosening and a healed greater trochanter fracture. A magnetic artifact reduction sequence-MRI of the right hip revealed peritrochanteric fluid suggestive of adverse local tissue reaction and possible pseudotumor formation without evidence of abductor destruction.

#### Intraoperative care

**Figure 7:**
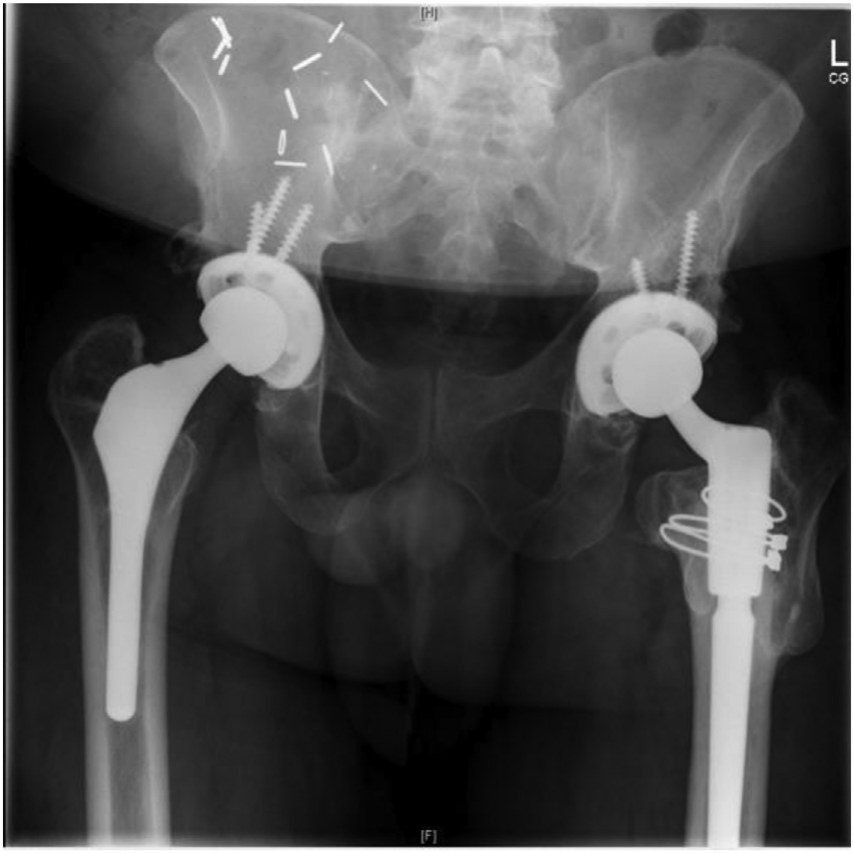
Postoperative radiographs for Patient 2-case 3, 2 years from right revision total hip arthroplasty.

He was then indicated for revision right THA. A standard posterior approach through the previous incision was taken. Black metal staining of the pericapsular tissue was appreciated and debrided. After hip dislocation, the cobalt chromium head was removed and minimal corrosion of the trunnion was appreciated. An acetabular explant system was used to debond the cup with minimal bone loss. A 62-mm Stryker Trident (Mahwah, NJ) revision cup was then impacted followed by 4 pelvic screws. A polyethylene liner was then placed followed by a 36-mm Biolox OPTION ceramic femoral head with a titanium sleeve adaptor from DJO Global (Austin, TX) (Fig. 7). The implant was stable on testing and the incision was then closed in standard fashion. Histology revealed polyethylene debris and metallic particles with a predominate macrophage mediated osteolysis. No significant lymphocytic infiltrate or findings consistent with infection were appreciated.

#### Postoperative care

His postoperative course was unremarkable and was subsequently discharged to subacute rehab. At his 2-year final follow-up, he was noted to be doing very well, pain controlled, and walking without assistive devices with radiographs that showed well-fixed implants without lucencies or malposition (Fig. 7).

## Discussion

Herein, we present 2 patients who received primary bilateral MoM-THA in the early 2000s that required revision arthroplasty due to ALTR less than 15 years later. The FMP acetabular component with a metal inlay polyethylene liner is another MoM articulation that is highly litigious. A brief online search of “Encore Hip Implant” results in numerous links to medical malpractice firms seeking compensation for patients with this implant design. It is imperative that the revision arthroplasty surgeon be familiar with this design and understand revision surgery may be accompanied with a lawsuit against the manufacturer.

The authors hypothesize failure of this unique implant occurred due to metal wear resulting in polyethylene wear leading to fracture of the polyethylene liner which caused the metal inlay to dissociate and abrade the femoral neck. The immune response to the polyethylene debris resulted in a macrophage mediated osteolysis as evidenced by the histology from case 2. As the polyethylene wear progressed to liner fracture and metal inlay dissociation causing femoral neck abrasion, an ALTR with pseudotumor formation occurred as evidenced by the MRI imaging and intraoperative findings of the initial revision surgery in case 1.

It is important for the surgeon to recognize this implant radiographically as a construct that can be associated with ALTR that may require revision to allow screening of asymptomatic patients that present in clinic. The FMP acetabular implant is often mated to the DJO-Encore Revelation femoral stem (Austin, TX) which has a unique distinguishable lateral flare which can be the first indication to evaluate for the presence of the metal inlay polyethylene. Additionally, the FMP acetabular shell is also flared at the rim which is also recognizable radiographically. Finally, the metal inlay is subtly recognizable surrounding the femoral head with a zone of lucency between the liner and shell where the polyethylene is located, which can be easily missed unless scrutinized closely. Recognizing these features radiographically is important to alert the surgeon to the possibility of early complications and to prepare the surgeon for challenges related to this implant during revision surgery.

First, the acetabular shell is not a true hemisphere due to the lateral flare which makes acetabular explantation with hemispherical cutting blades difficult causing excessive retro-acetabular bone loss and leading to over medialization of the acetabulum. It can be helpful to cut a shallow trough around the acetabular shell with curved gouges so it is easier for the hemispherical cutting blades to get started. Additionally, it may be helpful to choose an initial short cutting blade that is 1 mm larger than the outer diameter of the implant to clear the lateral flare prior to switching to a longer blade that matches the outer diameter off the shell to complete the removal. The femoral heads associated with the FMP acetabular system are uniquely sized at 34 mm, and many hemispherical cutting guides do not have a matching size. In the event the liner has not spun out of the shell, using a 32-mm femoral head on the acetabulum can be helpful. If acetabular screws are present, the metal liner can be removed by prying it out of the polyethylene liner with a 1/4 inch curved osteotome and mallet. The polyethylene liner can then be removed with either the tap and screw technique or sectioning with a helical burr to gain access to screws.

The FMP acetabular system is mated to the Encore/DJO Revelation stem (Austin, TX) which has a well born out track record with 15-year follow-up studies reporting zero cases of femoral component aseptic revision [10]. The stem is designed with a lateral flare to increase loading through the greater trochanter which is thought to better mimic the physiologic loading of the medial and lateral proximal femur [11]. During revision surgery, it is important to recognize this lateral flare preoperatively and understand the significant bony ingrowth that occurs within the greater trochanter. Intraoperatively the trunnion should be examined to determine if the stem is compromised or salvageable. If the stem is salvageable, it is helpful to have recognized the implant preoperatively as the femoral head sizes are uniquely 34 mm, which allows the surgeon to contact the manufacturer to obtain a manufacturer specific femoral head or obtain a titanium sleeve adaptor. If the stem requires revision due to catastrophic failure or even significant taper damage, the surgeon should give strong consideration to performing an extended trochanteric osteotomy to prevent an iatrogenic femur fracture of the greater trochanter during stem removal due to the extensive ingrowth within the greater trochanter.

The FMP acetabular system (Austin, TX) is still being manufactured, but the metal inlay polyethylene liner is not. It is important to note that midterm failure is not a problem that is being encountered with the other shells and articulation combinations available in this system.

There are only a few larger cohort studies evaluating MoM-THA with metal inlay polyethylene liners. However, none of those larger cohort studies include the metal inlay polyethylene from the FMP acetabular system. There are reports of the Metasul (Zimmer, Warsaw, IN) MoM-THA with a metal inlay polyethylene liner in patients younger than 50 years at the 10- and 15-year minimum follow-up that have found a found a 99% survivorship at 13 years for aseptic revision and 93% survivorship for all cause revision at 18 years [6,8]. The Metasul system utilized a 28-mm head which may have led to better survivorship due to less volumetric wear as compared to larger head MoM bearings. Only 1 case report regarding revision of the FMP metal-on-polyethylene liner has been reported where revision was performed due to the acetabular liner dissociating and rotating into a vertical position causing impingement on the neck without gross evidence of catastrophic failure. The metal head and metal-inlay polyethylene liner were exchanged for a metal-on-polyethylene articulation and the femoral stem was retained, in contrast to our case [12].

## Summary

Herein, we report on 2 patients with unique metal inlay polyethylene liners who suffered from ALTR that required revision arthroplasty. In 2 of 3 cases, the femoral trunnion was noted to have minimal corrosion and the stem was salvaged with a titanium adapter. In 1 case, there was impending catastrophic femoral stem failure and an extended trochanteric osteotomy was required to remove the unique lateral flare stem which loads the greater trochanter and allows excellent ingrowth. The surgeon must pay special attention when scrutinizing radiographs to identify a metal inlay polyethylene liner and when performing femoral explantation to prevent greater trochanter fracture in a patient who likely already has compromised abductor function due to ALTR.

## Conflicts of interest

The authors declare there are no conflicts of interest.

For full disclosure statements refer to https://doi.org/10.1016/j.artd.2023.101106.

## Informed Patient Consent

The author(s) confirm that written informed consent has been obtained from the involved patient(s) or if appropriate from the parent, guardian, power of attorney of the involved patient(s); and, they have given approval for this information to be published in this case report (series).

## Appendix A Supplementary Data

Conflict of Interest Statement for Reddy

Conflict of Interest Statement for Zvi

Conflict of Interest Statement for Weiser
